# Experimental and Modeling Investigations of Miniaturization in InGaN/GaN Light-Emitting Diodes and Performance Enhancement by Micro-Wall Architecture

**DOI:** 10.3389/fchem.2020.630050

**Published:** 2021-01-26

**Authors:** Yiping Zhang, Shunpeng Lu, Ying Qiu, Jing Wu, Menglong Zhang, Dongxiang Luo

**Affiliations:** ^1^ School of Electrical and Electronic Engineering, Nanyang Technological University, Singapore, Singapore; ^2^ Guangdong Research and Design Center for Technological Economy, Guangzhou, China; ^3^ Institute of Semiconductors, South China Normal University, Guangzhou, China

**Keywords:** GaN, light-emitting diode, miniaturization, size effect, micro-LED, current crowding effect, thermal dissipation

## Abstract

The recent technological trends toward miniaturization in lighting and display devices are accelerating the requirement for high-performance and small-scale GaN-based light-emitting diodes (LEDs). In this work, the effect of mesa size-reduction in the InGaN/GaN LEDs is systematically investigated in two lateral dimensions (*x*- and *y-*directions: parallel to and perpendicular to the line where p-n directions are) both experimentally and numerically. The role of the lateral size-reduction in the *x-* and *y-*directions in improving LED performance is separately identified through experimental and modeling investigations. The narrowed dimension in the *x-*direction is found to cause and dominate the alleviated current crowding phenomenon, while the size-reduction in the *y-*direction has a minor influence on that. The size-reduction in the *y-*orientation induces an increased ratio of perimeter-to-area in miniaturized LED devices, which leads to improved thermal dissipation and light extraction through the sidewalls. The grown and fabricated LED devices with varied dimensions further support this explanation. Then the effect of size-reduction on the LED performance is summarized. Moreover, three-micro-walls LED architecture is proposed and demonstrated to further promote light extraction and reduce the generation of the Joule heat. The findings in this work provide instructive guidelines and insights on device miniaturization, especially for micro-LED devices.

## Introduction

Owing to advantages in reliability, long lifetime, vivid colors, and energy efficiency, InGaN/GaN multiple quantum well (MQW) light-emitting diodes (LEDs) are regarded as a promising candidate to replace conventional lighting devices and have been extensively applied in various areas, such as automotive, backlight sources, display screens, electronic equipment, communicating applications, and general lighting (Ponce and Bour, 1997; Pimputkar et al., 2009; Kobayashi et al., 2012; Han et al., 2013). In order to fulfill the requirement of these applications, LEDs with various sizes and geometries are developed and fabricated accordingly (Kim et al., 2013; Cai et al., 2018). The micro-LED display is an emerging technology in general lighting and displays technology, which has shown its advantages in low power consumption, high dynamic range, short response time, and high contrast ratio (Hwang et al., 2017). The micro-LED display utilizes existing LED technology, which is significantly more efficient at producing light compared to OLED, cathode ray tube (CRT), and other display technologies. The huge opportunity in consumer electronics and the increasing applications in virtual reality, wearable devices, augmented reality, and medical applications become the major driving force behind the recent rapidly growing development in mini- and micro-LEDs (Park et al., 2009; Scharf et al., 2016; Son et al., 2018; Roche, 2019; Tang et al., 2019; Zhang et al., 2020). Extensive efforts of research have been devoted to studying the influence of size-reduction in the GaN-based LEDs (Choi et al., 2003a; Sadaf et al., 2016; Kang et al., 2017; Wu et al., 2018; Huang et al., 2019; Wong et al., 2019; Lin and Jiang, 2020). Francois et al. reported the lower external quantum efficiency (EQE) and maximum EQE when the LED devices went smaller (Olivier et al., 2017). Anis et al. also suggested the stronger Shockley-Read-Hall (SRH) non-radiative recombination was caused when the LED size tended to diminish (Daami et al., 2018). However, Tao et al. pointed out that the strain in QW was relaxed and Auger recombination was suppressed for smaller size LEDs, which lead to improved performance (Tao et al., 2012). Besides, Bourim et al. indicated that the junction temperature of larger LED chips was higher than the smaller ones under the same current densities, which resulted in carrier escapes from quantum wells thus degraded performance was observed (Bourim and Han, 2016). Huang et al. further concluded in the recent work that micro-LED would gradually move toward the central stage of the future display due to its advantages in energy efficiency (Huang et al., 2020).

Despite extensive reports on the effect of size-reduction in the GaN-based LEDs, no conclusive remarks can be drawn. Therefore, a systematical study with complementary theoretical simulation and experimental investigation is strongly required to resolve the discrepancy and uncover the underlying physics. Furthermore, most of the previous works focus on studying the influence of the total size or shape of the lateral mesa on the LED performance without analyzing the individual influence of size-reduction in one certain dimension. Thus, in this work, the size-reduction effect on the performance of InGaN/GaN LEDs is systematically analyzed with both experimental and numerical investigations. The influence of the size-reduction of the lateral mesa in two directions on the performance of the LED chip is separately identified. Then the effect of size-reduction on the GaN-based LEDs is concluded, which offers instructive guidelines in device miniaturization. Finally, a three-micro-walls LED architecture is proposed and demonstrated with the aim of improving the light extraction efficiency and reducing the thermal heat generated.

## Materials and Methods

**FIGURE 1:**
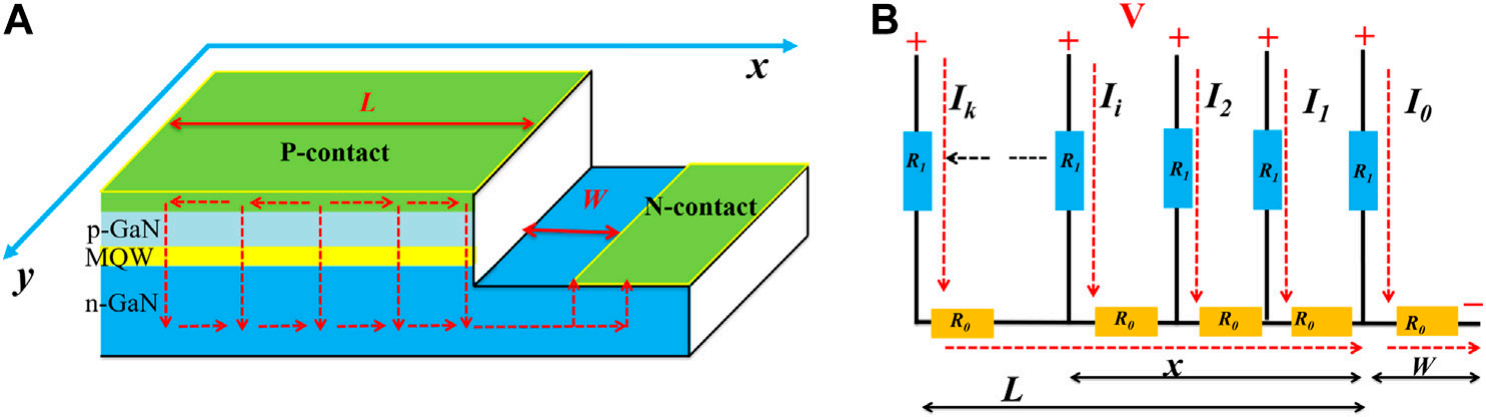
**(A)** The schematic diagram of the structure and the current flow paths from the p-GaN layer to the n-GaN layer, and **(B)** equivalent circuit of current path model for the flip-chip InGaN/GaN LEDs.

The InGaN/GaN MQW LEDs studied in this work were grown on c-plane patterned sapphire substrates by metal-organic chemical vapor deposition (MOCVD) system. The sapphire substrate used is the two-inch patterned-sapphire substrate that has periodic cone patterns with a diameter of 2.4 μm, a height of 1.5 μm, and a pitch of 3 μm. The epitaxial growth was initiated on a 30-nm thick low-temperature GaN nucleation layer followed by a 4 µm unintentionally doped n-type GaN (u-GaN) layer. Subsequently, a 2-μm thick Si-doped GaN layer was grown with a doping concentration of 5 × 10^18^ cm^−3^, in which SiH_4_ was adopted as the dopant source. Then, eight pairs of In_0.15_Ga_0.85_N/GaN MQWs with 3-nm thick QW and 12-nm thick QB were grown. In addition, a 20-nm thick p-doped Al_0.15_Ga_0.85_N electron blocking layer (EBL) was grown to suppress the excess electron overflow into the p-GaN region. Then a 200-nm thick Mg-doped GaN with a doping concentration of 3 × 10^17^ cm^−3^ was grown as the hole source layer. The p-type conductivity of the EBL and the hole source layer was realized by Mg doping where Cp_2_Mg was used as the Mg precursor. Subsequent to the epitaxial growth, the LED wafers were fabricated into flip-chip LED devices using standard fabrication processes. The mesa area was shaped using reactive ion etching (RIE) for LED devices of different sizes. Ni/Ag (5 nm/5 nm) metal layers were deposited as the current spreading layer using e-beam evaporation, and Ti/Au (30 nm/1,000 nm) metal layers were deposited as p- and n-electrode contact. A schematic diagram of the device structure is shown in Figure 1A. The current-voltage characteristics were determined by a LED tester (M2442S-9A Quatek Group) and the optical output power was measured by an integrating sphere attached to an Ocean Optics spectrometer (QE65000).

To study the physical mechanism on how the size-reduction of the lateral mesa influences the LED performance, numerical simulations were conducted by Advanced Physical Models of Semiconductor Devices (APSYS) simulator, which self-consistently solves the Schrödinger equation, continuity equation, and Poisson equation with proper boundary conditions. In the simulations, the Auger recombination coefficient was set to 1 × 1,042 m^6^/s and 40% of the polarization charges were assumed such that 60% of the theoretical polarization charges were released because of the crystal strain relaxation by generating dislocations. The other parameters used in the simulation can be found elsewhere (Meneghini et al., 2009; Kim et al., 2010; Kuo et al., 2011; Park et al., 2013; Zhang et al., 2013; Zhang et al., 2017).

## Results and Discussion

Figure 1A presents the schematic diagram of the structure and the current flow paths for the flip-chip InGaN/GaN LEDs, which is extensively used in commercial products and academic research due to its superiority in light extraction. In order to eliminate the influence of the electrode pattern (eg fingers), the p-contact metal layer covers the entire top surface of the p-GaN layer. As shown in Figure 1A, there are two lateral orientations to follow when reducing the mesa size of the LED device: *x-* and *y-*directions. In order to identify the individual influence on the LED performance, the effect of size-reduction in these two lateral orientations is separately investigated with the *x-*direction as the beginning. As we can see from the equivalent circuit shown in Figure 1B, there are plenty of current paths from the p-GaN layer to the n-GaN layer and according to Ohm's law we will have:

$$I_{i}=\frac{V}{R_{1}+x_{i}R_{0}+WR_{0}},$$ (1)

where *I*_*i*_ is current corresponding to each path where *i* = 1, 2, …, *k*−1, and *k*, in which *k* is infinity, *x*_*i*_ denotes its corresponding length, *V* is the bias voltage applied to the LED, *R*_*1*_ denotes the resistance along the vertical direction from the p-contact layer to the active region, *L* is the mesa size in the *x*-direction, *W* is the lateral length from the edge of n-contact to the edge of the p-GaN layer, and *R*_*0*_ is the resistance per unit length of the n-GaN layer in the lateral direction along *x*. Then the average current for all current paths is obtained, as shown below:

$$I_{\text{average}}=\frac{1}{L}\overset{L}{\underset{0}{\int }}I_{i}dx=\frac{1}{L}\overset{L}{\underset{0}{\int }}\frac{V}{R_{1}+x_{i}R_{0}+WR_{0}}dx,$$ (2)

$$I_{\text{average}}=\frac{V}{R_{0}L}\left(\ln\frac{1}{R_{1}+LR_{0}+WR_{0}}-\ln\frac{1}{R_{1}+WR_{0}}\right).$$ (3)

As we can see from the above equation, the average current for all the current paths is increased with the reduction of mesa size in the *x*-direction, which means the current becomes more uniform in the lateral direction and thus the current crowding effect is alleviated. As the p-GaN layer is entirely covered by the p-contact metal, the reduction of the mesa size in the *y-*direction has a minor contribution in alleviating the current crowding effect. Therefore, the alleviated current crowding as a consequence of the narrowing in the mesa size mainly originates from the uniform current spreading in the lateral *x-*direction. It is worth noting that if the finger shape or other types of electrode pattern is adopted in the p-contact metal layer, the electrode distribution along the lateral directions (both *x*- and *y*-directions) will further affect the current spreading performance. In that case, the current spreading is subjected to the coupling effect of size-reduction in the *x-*direction and electrode patterns (Guo and Schubert, 2001; Song, 2012).

**FIGURE 2:**
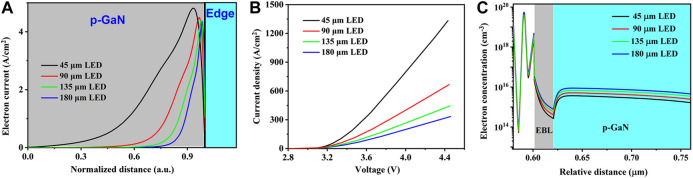
**(A)** The simulated horizontal profiles of electron current density along the *x-*direction in the p-GaN layer as a function of normalized distance, **(B)** calculated *J*-*V* curves, and **(C)** computed electron concentration in the EBL and the p-GaN region at the current density of 300 A/cm^2^ for LEDs with varied mesa dimensions of 45 × 200, 90 × 200, 135 × 200, and 180 × 200 μm^2^, respectively.

In order to further support the above conclusion that the size-reduction in the *x-*direction contributes to alleviated current crowding, the numerical calculations are conducted for the InGaN/GaN MQW LEDs with varied device dimensions of 45 × 200, 90 × 200, 135 × 200, and 180 × 200 μm^2^, respectively. Here, the mesa dimension in the *x*-direction is narrowed from 180 to 45 μm, while the dimension in the *y*-direction is fixed at 200 μm. Figure 2A shows horizontal profiles of electron current densities in the p-GaN layer for the LEDs with different mesa dimensions. Since the LEDs are of different mesa dimensions, the electron distribution along the *x-*direction in Figure 2A is normalized by dividing the corresponding dimension (45, 90, 135, 180 μm). It can be clearly seen that most electrons are located at the edge of the p-electrode for all the LEDs with varied mesa sizes, implying the inhomogeneous distribution of carrier in the *x-*direction. This is called the current crowding effect, which is always a tough issue for lateral LED geometry. However, the electrons distribute more uniform along the *x-*direction for smaller LEDs, as presented in Figure 2A, which indicates alleviated current crowding. The improved performance in the narrowed LEDs can also be observed in the curves for current density (*J*) vs. voltage (*V*) as shown in Figure 2B, which is attributed to the uniform current spreading and consistent with the results in the current path model and previous reports (Tian et al., 2012; Yang et al., 2014; Bourim and Han, 2016).

Moreover, owing to the improved current spreading, the electron overflow into the p-GaN layer is suppressed. The simulated electron concentration near the p-GaN layer is depicted in Figure 2C for the LED devices with varied dimensions under the current injection level of 300 A/cm^2^, which demonstrates that the electron concentration in EBL and the p-GaN region is reduced when the mesa dimension decreases from 180 to 45 μm. The reduction of the electron concentration in the p-doped region indicates the effective suppression of the electron leakage. These results suggest that with a narrowed mesa dimension in the *x*-direction, the current crowding phenomenon is alleviated and the electrons will spread more homogeneously.

Based on the above analysis, the conclusion can be drawn that the mesa size-reduction of the InGaN/GaN LEDs in the lateral *x-*orientation leads to alleviated current crowding thus improved performance is obtained, while the reduction in the *y-*direction makes a minor contribution to the current spreading performance. However, the diminished dimension in the *y-*direction increases the ratio of perimeter-to-area. The increased ratio of perimeter-to-area in the smaller LEDs leads to improved heat dissipation and reduced self-heating, hence the temperature in the LED device is decreased (Kim et al., 2012; Horng et al., 2015). Moreover, the light extraction through the sidewalls is improved with diminished LED size, which contributes to the improvement of the LED performance (Stark et al., 2011).

**FIGURE 3:**
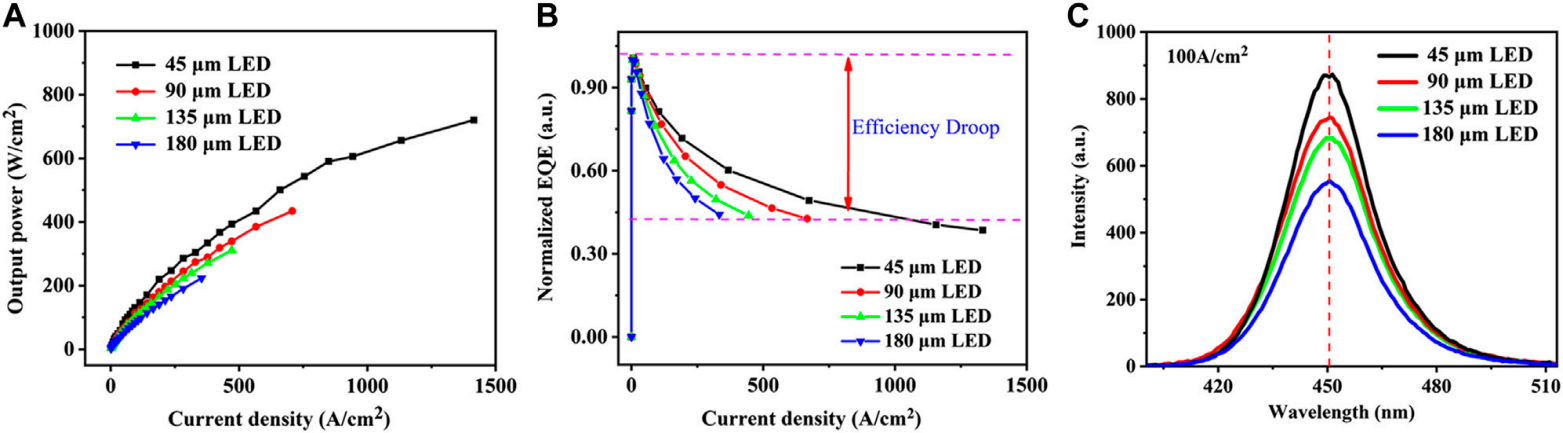
**(A)** Experimentally measured optical output power as a function of forward current density, **(B)** normalized EQE as a function of forward current density, and **(C)** electroluminescent spectral curves under the current density of 100 A/cm^2^ for LEDs with different dimensions of the mesa.

In order to confirm the above conclusion, InGaN/GaN LED chips with different sizes are fabricated for investigation with the dimensions of 200 × 45, 200 × 90, 200 × 135, and 200 × 180 μm^2^, respectively, which have the perimeter-to-area ratio of 0.54, 0.32, 0.25, and 0.21, respectively. Here, the mesa dimension in the *y*-direction is narrowed from 180 to 45 μm, while the dimension in the *x*-direction is fixed at 200 μm. All LED chips are fabricated within a 2 mm × 2 mm region on the same epitaxial wafer to avoid the spatial inhomogeneity in the wafer with standard fabrication procedures in order to eliminate the influence by crystalline quality, epitaxial growth, and fabrication processes, which is the possible reason for the discrepancy in the conclusion of the size-reduction effect among the previous reports (Tao et al., 2012; Olivier et al., 2017; Singh et al., 2017; Daami et al., 2018). The p-contact metal totally covers the top surface of the p-GaN layer to avoid the influence of the electrode pattern, which is different from the previous works (Song, 2012; Singh et al., 2017). The experimentally measured optical output power as a function of current density for LEDs with different mesa dimensions are illustrated in Figure 3A, from which we can see that the optical output power increases consistently with the mesa size narrowed from 180 to 45 μm in the *y*-direction. The efficiency droop indicated in Figure 3B is also reduced for the narrowed LED devices, which is consistent with the observation in the previous report (Tao et al., 2012). The variation in the ending point of data shown in Figures 3A,B is because the optical output power and current are measured under the same biased voltage for LEDs with varied sizes. The enhancement in the performance observed in the smaller LED devices is attributed to the improved light extraction in the sidewall and improved heat dissipation due to the higher perimeter-to-area ratio and reduced device temperature (Choi et al., 2003a; Choi et al., 2003b). Figure 3C presents the electroluminescence (EL) spectra curves for the studied LEDs with different mesa dimensions, where the emission density is the strongest for the 45-μm LED. Meanwhile, the EL intensity for LEDs with larger mesa size is reduced as the increase of mesa dimension. The peak wavelength is about 450 nm and is not shifted when the size is reduced from 180 to 45 μm, as indicated in Figure 3C. However, it is worth noting that when the LED size is further reduced to the nanoscale, the blue shift in the EL curve can be anticipated due to reduced energy band tilting caused by the strain relaxation in the smaller LED device (Demangeot et al., 2002; Wu et al., 2008; Stark et al., 2011).

Thereafter, we can conclude the effect of size-reduction (*E*) for InGaN/GaN LEDs as following:

E = C(x) + P(x, y) + T(x, y) + S(x, y) − N(x,y) (4)

where *C*(*x*) is the alleviated current crowding effect (Ge et al., 2019), *P* (*x*, *y*) is the improvement achieved by the increased perimeter-to-area ratio, including improved thermal dissipation and light extraction (Choi et al., 2003a; Choi et al., 2003b), *T* (*x*, *y*) is the reduced self-heating and improved heat distribution (Gong et al., 2010; Ploch et al., 2013), *S* (*x*, *y*) is the enhancement owing to the strain relaxation (Demangeot et al., 2002; Tao et al., 2012; Ge et al., 2019), and *N* (*x*,*y*) is the possible negative effect due to the increased nonradiative recombination (Gong et al., 2010; Stark et al., 2011), surface recombination (Jin et al., 2001), and current leakage induced by etching damage and impurities during fabrications (Jin et al., 2000; Stark et al., 2011). A more detailed quantitative analysis of the above factors is needed to determine the dominant ones and great precautions must be undertaken to avoid degradation in devices during the growth and fabrication processes for smaller LEDs.

**FIGURE 4:**
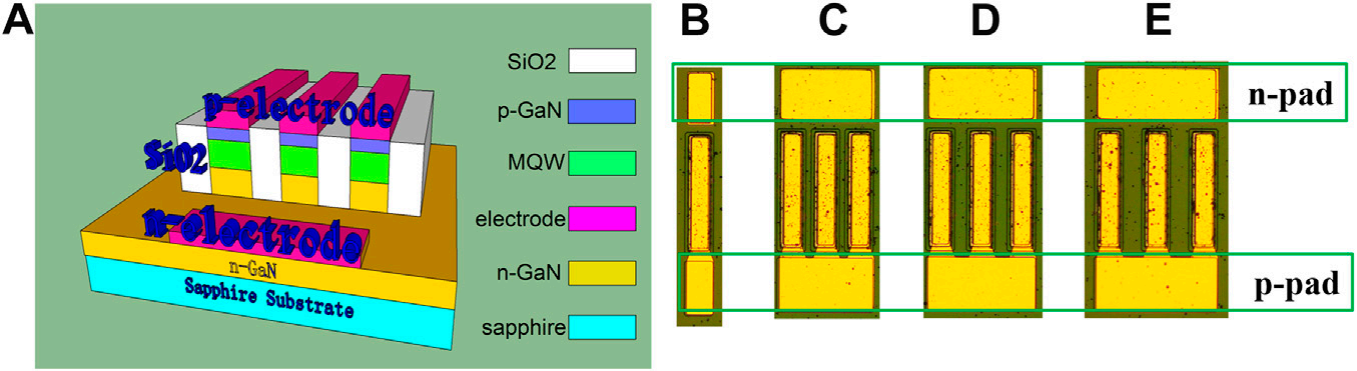
**(A)** Schematic diagram of the proposed LED architecture with three-micro-walls, and the fabricated LED devices of **(B)** single-micro-wall as controlled group, **(C)** three-micro-walls with wall spacing of 11 μm, **(D)** three-micro-walls with wall spacing of 22 μm, and **(E)** three-micro-walls with wall spacing of 33 μm.

In order to further improve the light extraction in the smaller InGaN/GaN LEDs, we design a three-micro-walls LED architecture by incorporating three micro-LEDs into a device. The schematic diagram of the proposed LED structure is shown in Figure 4A, in which the micro-walls are defined with the wall dimensions of 200 × 45 μm^2^. In order to reduce the current leakage and increase the light extraction efficiency, the wall gap is filled up with SiO_2_ by chemical vapor deposition (CVD), which has a refractive index of 1.47. For the proposed LEDs with three-micro-walls geometry, the wall spacing is designed to be varied as 11, 22, and 33 μm, and the corresponding fabricated LED devices are presented in Figures 4C–E, respectively. The single-micro-wall LED device is also introduced and fabricated as the controlled group, as shown in Figure 4B.

**FIGURE 5:**
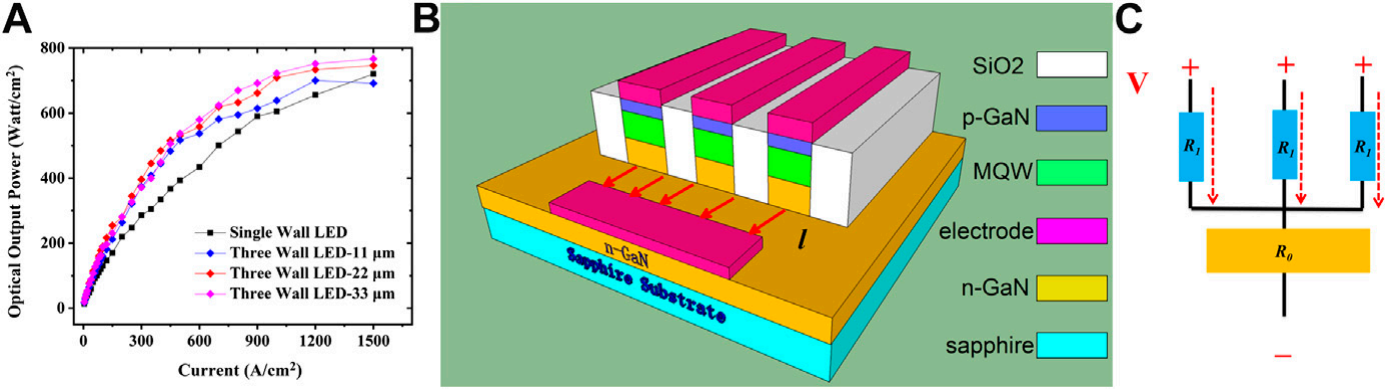
**(A)** Experimentally measured optical output power for proposed three-micro-walls LED devices and single-wall device, **(B)** illustration of the current path for proposed LED device, and **(C)** corresponding simplified current path model.

As the three-micro-walls LED device is three times the size of the single-wall LED, the optical power of the former is divided by three for comparison. The experimentally measured optical output power of the devices is shown in Figure 5A, in which all the proposed three-micro-walls LED devices perform better than the controlled group due to the improved light extraction through sidewalls achieved by the filling up of SiO_2_ in the gap. Meanwhile, the proposed architecture with the wall spacing of 33 μm has the best performance because more light escapes from the sidewall with a larger gap between the micro-walls. More importantly, there is less thermal heat generation for the LED device with wider wall spacing due to the reduced series resistance when current horizontally passes the n-GaN layer. Figures 5B,C depict the current paths and the corresponding simplified current paths model for the LEDs with three-micro-walls geometry. As indicated in Figure 5C, the Joule heat (*J*_*heat*_) generated in the LED can be expressed as:

$$J_{\text{heat}}\;=\;I^{2}\left(R_{1}\;+\;R_{0}\right)\;=\;I^{2}\left(R_{1}\;+\;p\frac{l}{S}\right),$$ (5)

where *R*_1_ is the total resistance from top contact layer to active region in the vertical direction, *R*_0_ denotes the resistance of n-GaN layer, *I* is current, *p* denotes the resistivity of n-GaN layer, *l* is the lateral distance from n-electrode to mesa edge, and *S* is the cross-sectional area of the n-GaN layer perpendicular to the direction of *l*. According to Eq. 5, with the increasing spacing of the micro-walls, the resistance of *R*_0_ increases due to the enlarged area *S*. Hence, the total heat generation in the LED device is reduced, which contributes to improved LED performance. It is worth noting that with a larger gap, the thermal heat dissipation is improved accordingly. Thus, the proposed LED architecture has superior performance due to improved light extraction, less thermal heat generation, and better heat dissipation.

## Conclusion

The InGaN/GaN LEDs with different mesa dimensions are investigated both experimentally and numerically to explore the size-reduction effect on the LED performance. The individual role of the size-reduction in the lateral *x-* and *y-*directions is identified separately. The physical mechanism of performance enhancement by mesa size-reduction is revealed by using a model of current paths, which suggests that the current crowding effect is alleviated when the lateral mesa size is narrowed in the *x-*direction. The calculated results indicate that the LED devices with narrowed dimensions in the *x*-direction have reduced electron leakage and a better current-voltage characteristic under the same current density, which is attributed to the effectively improved current spreading. Backed by the experiments, the decrease of the mesa size in the *y-*direction is found to improve the LED performance, which is owing to a higher ratio of perimeter-to-area, better thermal dissipation, and improved light extraction. Then the effect of size-reduction on the LED performance is concluded. Finally, a new LED architecture with three-micro-walls is proposed and demonstrated. The proposed three-micro-walls LEDs are observed to have improved optical performance compared to the controlled group and with the increase of wall spacing, the improvement becomes conspicuous, which is owing to the increased light extraction and less Joule heat generation resulting from the reduced series resistance when current horizontally passes the n-GaN layer.

## Data Availability Statement

The original contributions presented in the study are included in the article/Supplementary Material, further inquiries can be directed to the corresponding authors.

## Author Contributions

All authors have made a substantial, direct, and intellectual contribution to the work and approved it for publication.

## Conflict of Interest

The authors declare that the research was conducted in the absence of any commercial or financial relationships that could be construed as a potential conflict of interest.

## References

[B1] Bourim E.-M. Han J. I. (2016). Size effect on negative capacitance at forward bias in InGaN/GaN multiple quantum well-based blue LED. Electron. Mater. Lett. 12, 67–75. 10.1007/s13391-015-5281-9

[B2] Bruchas Y. Kumakura K. Akasaka T. Makimoto T. (2012). Layered boron nitride as a release layer for mechanical transfer of GaN-based devices. Nature 484, 223–227. 10.1038/nature10970 22498627

[B3] Cai Y. Zou X. Liu C. Lau K. M. (2018). Voltage-controlled GaN HEMT-LED devices as fast-switching and dimmable light emitters. IEEE Electron. Device Lett. 39, 224–227. 10.1109/led.2017.2781247

[B4] Chen M. S. Nakamura S. Denbaars S. P. (2019). Review—progress in high performance III-nitride micro-light-emitting diodes. ECS Journal of Solid State Science and Technology 9, 015012 10.1149/2.0302001jss

[B5] Choi H. W. Jeon C. W. Dawson M. D. Edwards P. R. Martin R. W. (2003a). Fabrication and performance of parallel-addressed InGaN micro-LED arrays. IEEE Photon. Technol. Lett. 15, 510–512. 10.1109/lpt.2003.809257

[B6] Choi H. W. Jeon C. W. Dawson M. D. Edwards P. R. Martin R. W. Tripathy S. (2003b). Mechanism of enhanced light output efficiency in InGaN-based microlight emitting diodes. J. Appl. Phys. 93, 5978–5982. 10.1063/1.1567803

[B7] Daami A. Olivier F. Dupré L. Henry F. Templier F. (2018). 59-4: invited Paper: electro-optical size-dependence investigation in GaN micro-LED devices. SID Symp. Digest Tech. Papers 49, 790–793. 10.1002/sdtp.12325

[B8] Demangeot F. Gleize J. Frandon J. Renucci M. A. Kuball M. Peyrade D. (2002). Optical investigation of micrometer and nanometer-size individual GaN pillars fabricated by reactive ion etching. J. Appl. Phys. 91, 6520–6523. 10.1063/1.1468908

[B9] Gong Z. Jin S. Chen Y. Mckendry J. Massoubre D. Watson I. M. (2010). Size-dependent light output, spectral shift, and self-heating of 400 nm InGaN light-emitting diodes. J. Appl. Phys. 107, 013103 10.1063/1.3276156

[B10] Guo X. Schubert E. F. (2001). Current crowding and optical saturation effects in GaInN/GaN light-emitting diodes grown on insulating substrates. Appl. Phys. Lett. 78, 3337–3339. 10.1063/1.1372359

[B11] Han N. Cuong T. Han M. Ryu B. Chandramohan S. Park J. (2013). Improved heat dissipation in gallium nitride light-emitting diodes with embedded graphene oxide pattern. Nat. Commun. 4, 1452 10.1038/ncomms2448 23385596

[B12] Hong R.-H. Chen K.-Y. Tien C.-H. Liao J.-C. (2015). Effects of mesa size on current spreading and light extraction of GaN-based LEDs. J. Disp. Technol. 11, 1010–1013. 10.1109/jdt.2015.2461015

[B13] Huang Chen Y.-R. Yu P. Chiu C. Chang C.-Y. Kuo H. (2008). “Analysis of strain relaxation and emission spectrum of a free-standing GaN-based nanopillar,” in Eighth International Conference on Solid State Lighting, San Diego, CA (London: SPIE).

[B14] Huang Y. Hsiang E.-L. Deng M.-Y. Wu S.-T. (2020). Mini-LED, Micro-LED and OLED displays: present status and future perspectives. Light Sci. Appl. 9, 105 10.1038/s41377-020-0341-9 32577221 PMC7303200

[B15] Huang Y. Tan G. Gou F. Li M.-C. Lee S.-L. Wu S.-T. (2019). Prospects and challenges of mini-LED and micro-LED displays. J. Soc. Inf. Disp. 27, 387–401. 10.1002/jsid.760

[B16] Hwang D. Mughal A. Pynn C. D. Nakamura S. Denbaars S. P. (2017). Sustained high external quantum efficiency in ultrasmall blue III–nitride micro-LEDs. APEX 10, 032101 10.7567/apex.10.032101

[B17] Jin S. X. Li J. Li J. Z. Lin J. Y. Jiang H. X. (2000). GaN microdisk light emitting diodes. Appl. Phys. Lett. 76, 631–633. 10.1063/1.125841

[B18] Jin S. X. Shakya J. Lin J. Y. Jiang H. X. (2001). Size dependence of III-nitride microdisk light-emitting diode characteristics. Appl. Phys. Lett. 78, 3532–3534. 10.1063/1.1376152

[B19] Kang C. M. Kong D. J. Shim J. P. Kim S. Choi S. B. Lee J. Y. (2017). Fabrication of a vertically-stacked passive-matrix micro-LED array structure for a dual color display. Optic Express 25, 2489–2495. 10.1364/OE.25.002489 29519094

[B20] Kim T. I. Jung Y. H. Song J. Kim D. Li Y. Kim H. S. (2012). High-efficiency, microscale GaN light-emitting diodes and their thermal properties on unusual substrates. Small 8, 1643–1649. 10.1002/smll.201200382 22467223

[B21] Kuo Y.-K. Wang T.-H. Chang J.-Y. Tsai M.-C. (2011). Advantages of InGaN light-emitting diodes with GaN-InGaN-GaN barriers. Appl. Phys. Lett. 99, 091107 10.1063/1.3633268

[B22] Lee K. S. Kim J. H. Jung S. J. Park Y. J. Cho S. N. (2010). Stable temperature characteristics of InGaN blue light emitting diodes using AlGaN/GaN/InGaN superlattices as electron blocking layer. Appl. Phys. Lett. 96, 091104 10.1063/1.3340939

[B23] Lin J. Y. Jiang H. X. (2020). Development of microLED. Appl. Phys. Lett. 116, 100502 10.1063/1.5145201

[B24] Manin-Ferlazzo C. Li J. Wang G. Su K. Lu X. (2019). Size effect on optical performance of blue light-emitting diodes. J. Semiconduct. 40, 102301 10.1088/1674-4926/40/10/102301

[B25] Meneghini M. Trivellin N. Meneghesso G. Zanoni E. Zehnder U. Hahn B. (2009). A combined electro-optical method for the determination of the recombination parameters in InGaN-based light-emitting diodes. J. Appl. Phys. 106, 114508 10.1063/1.3266014

[B26] Olivier F. Tirano S. Dupré L. Aventurier B. Largeron C. Templier F. (2017). Influence of size-reduction on the performances of GaN-based micro-LEDs for display application. J. Lumin. 191, 112–116. 10.1016/j.jlumin.2016.09.052

[B27] Park J. H. Kim D. Y. Hwang S. Meyaard D. Schubert E. F. Han Y. D. (2013). Enhanced overall efficiency of GaInN-based light-emitting diodes with reduced efficiency droop by Al-composition-graded AlGaN/GaN superlattice electron blocking layer. Appl. Phys. Lett. 103, 061104 10.1063/1.4817800

[B28] Park S. I. Xiong Y. Kim R. H. Elvikis P. Meitl M. Kim D. H. (2009). Printed assemblies of inorganic light-emitting diodes for deformable and semitransparent displays. Science 325, 977–981. 10.1126/science.1175690 19696346

[B29] Ploch N. L. Rodriguez H. Stolmacker C. Hoppe M. Lapeyrade M. Stellmach J. (2013). Effective thermal management in ultraviolet light-emitting diodes with micro-LED arrays. IEEE Trans. Electron. Dev. 60, 782–786. 10.1109/ted.2012.2234462

[B30] Ponce F. A. Bour D. P. (1997). Nitride-based semiconductors for blue and green light-emitting devices. Nature 386, 351–359. 10.1038/386351a0

[B31] Roche E. T. (2019). Implanted device enables responsive bladder control. Nature 565, 298–300. 10.1038/d41586-018-07811-1 30643302

[B32] Rogers S. Speck J. S. Denbaars S. P. Nakamura S. (2009). Prospects for LED lighting. Nat. Photon. 3, 180–182. 10.1038/nphoton.2009.32

[B33] Rogers T. I. Mccall J. G. Jung Y. H. Huang X. Siuda E. R. Li Y. (2013). Injectable, cellular-scale optoelectronics with applications for wireless optogenetics. Science 340, 211–216. 10.1126/science.1232437 23580530 PMC3769938

[B34] Sadaf S. M. Ra Y. H. Szkopek T. Mi Z. (2016). Monolithically integrated metal/semiconductor tunnel junction nanowire light-emitting diodes. Nano Lett. 16, 1076–1080. 10.1021/acs.nanolett.5b04215 26812264

[B35] Scharf R. Tsunematsu T. Mcalinden N. Dawson M. D. Sakata S. Mathieson K. (2016). Depth-specific optogenetic control *in vivo* with a scalable, high-density μLED neural probe. Sci. Rep. 6, 28381 10.1038/srep28381 27334849 PMC4917834

[B36] Singh S. Kumar S. Pal S. Dhanavantri C. (2017). Performances of p-side down vertical InGaN/GaN blue light-emitting diodes with chip size. Optic Laser. Technol. 95, 165–171. 10.1016/j.optlastec.2017.05.002

[B37] Son K. R. Lee T. H. Lee B. R. Im H. S. Kim T. G. (2018). Nitride-based microlight-emitting diodes using AlN thin-film electrodes with nanoscale indium/tin conducting filaments. Small 14, 1801032 10.1002/smll.201801032 30286283

[B38] Song X. Zeng J. Jin Y. Meng X. (2012). Optoelectronic properties of GaN-based light-emitting diodes with different mesa structures. Mater. Sciences Appl. 03, 838–842. 10.4236/msa.2012.312122

[B39] Stark C. J. M. Detchprohm T. Wetzel C. (2011). The role of mesa size in nano-structured green AlGaInN light-emitting diodes. Phys. Status Solidi C 8, 2311–2314. 10.1002/pssc.201001190

[B40] Tang B. Miao J. Liu Y. Wan H. Li N. Zhou S. (2019). Enhanced light extraction of flip-chip mini-LEDs with prism-structured sidewall. Nanomaterials 9, 319 10.3390/nano9030319 PMC6473491 30823374

[B41] Tao Y. B. Wang S. Y. Chen Z. Z. Gong Z. Xie E. Y. Chen Y. J. (2012). Size effect on efficiency droop of blue light emitting diode. Phys. Status Solidi C 9, 616–619. 10.1002/pssc.201100483

[B42] Wu T. Sher C.-W. Lin Y. Lee C.-F. Liang S. Lu Y. (2018). Mini-LED and micro-LED: promising candidates for the next generation display technology. Appl. Sci. 8, 1557 10.3390/app8091557

[B43] Yang W. Zhang S. Mckendry J. J. D. Herrnsdorf J. Tian P. Gong Z. (2014). Size-dependent capacitance study on InGaN-based micro-light-emitting diodes. J. Appl. Phys. 116, 044512 10.1063/1.4891233

[B44] Zhang K. Liu Y. Kwok H.-S. Liu Z. (2020). Investigation of electrical properties and reliability of GaN-based micro-LEDs. Nanomaterials 10, 689 10.3390/nano10040689 PMC7221619 32268479

[B45] Zhang P. Mckendry J. J. D. Gong Z. Guilhabert B. Watson I. M. Gu E. (2012). Size-dependent efficiency and efficiency droop of blue InGaN micro-light emitting diodes. Appl. Phys. Lett. 101, 231110 10.1063/1.4769835

[B46] Zhang Y. Zhang Z.-H. Tan S. T. Hernandez-Martinez P. L. Zhu B. Lu S. (2017). Investigation of p-type depletion doping for InGaN/GaN-based light-emitting diodes. Appl. Phys. Lett. 110, 033506 10.1063/1.4973743

[B47] Zhang Z.-H. Tiam Tan S. Kyaw Z. Ji Y. Liu W. Ju Z. (2013). InGaN/GaN light-emitting diode with a polarization tunnel junction. Appl. Phys. Lett. 102 10.1063/1.4806978

